# Correcting for Differential Transcript Coverage Reveals a Strong Relationship between Alternative Splicing and Organism Complexity

**DOI:** 10.1093/molbev/msu083

**Published:** 2014-03-27

**Authors:** Lu Chen, Stephen J. Bush, Jaime M. Tovar-Corona, Atahualpa Castillo-Morales, Araxi O. Urrutia

**Affiliations:** ^1^Department of Biology and Biochemistry, University of Bath, Bath, United Kingdom

**Keywords:** organism complexity, alternative splicing, genome evolution, transcriptome evolution, expressed sequence tags

## Abstract

What at the genomic level underlies organism complexity? Although several genomic features have been associated with organism complexity, in the case of alternative splicing, which has long been proposed to explain the variation in complexity, no such link has been established. Here, we analyzed over 39 million expressed sequence tags available for 47 eukaryotic species with fully sequenced genomes to obtain a comparable index of alternative splicing estimates, which corrects for the distorting effect of a variable number of transcripts per species—an important obstacle for comparative studies of alternative splicing. We find that alternative splicing has steadily increased over the last 1,400 My of eukaryotic evolution and is strongly associated with organism complexity, assayed as the number of cell types. Importantly, this association is not explained as a by-product of covariance between alternative splicing with other variables previously linked to complexity including gene content, protein length, proteome disorder, and protein interactivity. In addition, we found no evidence to suggest that the relationship of alternative splicing to cell type number is explained by drift due to reduced *N*_e_ in more complex species. Taken together, our results firmly establish alternative splicing as a significant predictor of organism complexity and are, in principle, consistent with an important role of transcript diversification through alternative splicing as a means of determining a genome’s functional information capacity.

## Introduction

Prior to widespread genome sequencing, it was assumed that organism complexity was proportional to gene content—that more complex organisms encode a greater amount of genetic information (Taft and Mattick 2003), the unit of which is the gene (Bird 1995). However, the sequencing of the human genome, revealing a lower than expected number of genes (Fields et al. 1994), initiated a hunt to uncover the genomic basis of organism complexity (Nilsen and Graveley 2010) as, despite two rounds of whole genome duplication at the base of the vertebrate lineage (Ohno 1970; Dehal and Boore 2005), the human genome contains almost as many genes as that of a worm (Lander et al. 2001). Several genomic features have been shown to have a significant association with organism complexity, measured as the number of distinct cell types per species (cell type number [CTN]). These variables include various measures of the potential number of molecular interactions per protein: the number and proportion of protein–protein interaction (PPI) domains in each protein (Xia et al. 2008; Schad et al. 2011) and protein disorder (flexibility in a protein’s 3D structure to adopt a variety of conformations) (Romero et al. 2006; Dunker et al. 2008; Schad et al. 2011). More recently, total coding region length in a genome was shown to be positively associated with organism complexity (Schad et al. 2011). This same study also showed that when restricting the analysis to metazoans, gene number becomes a significant predictor of organism complexity.

Alternative splicing, a posttranscriptional process in eukaryotes by which multiple distinct transcripts are produced from a single gene, has the potential to boost the total number of distinct proteins encoded in a genome in the absence of increases in gene number (Nilsen and Graveley 2010). As such, an association between alternative splicing and organism complexity has long been proposed. Under an “adaptive" model, an increase in alternative splicing could facilitate the evolution of higher organismal complexity, by increasing proteome diversity (and thus, diversifying functionality) at a level disproportionate to increases in the number of protein-coding genes (Graveley 2001; Xing and Lee 2007; Chen et al. 2012). Indeed, over the last decade, alternative splicing prevalence (ASP; the proportion of multiexon genes that have at least one alternative splicing event) has been successively revised upward for humans, with recent deep sequencing transcriptome analyses estimating that up to 94% of multiexon genes undergo alternative splicing (Pan et al. 2008; Wang et al. 2008). However, assessing the expansion of ASP through evolutionary time and establishing a link between alternative splicing and organism complexity have proved difficult (Nilsen and Graveley 2010). The main barrier to comparative studies of ASP arises from the fact that differences in transcript sequence coverage across species can distort both the proportion of genes classified as undergoing alternative splicing and the number of alternative splicing events detected (Brett et al. 2002; Kim et al. 2004; Kim et al. 2007; Takeda et al. 2008; Mollet et al. 2010; Nilsen and Graveley 2010; Schad et al. 2011). Kim et al. (2007) devised a method of transcript number normalization to obtain comparable ASP indices involving the identification of alternative splicing events from a random sample of 10 transcripts per gene. Importantly, they showed that alternative splicing in vertebrate species was higher than among invertebrates and that this was not explained by the higher abundance of transcripts available for vertebrate species. Although not directly tested, these findings were suggestive of a link between alternative splicing and complexity as vertebrates are generally considered to have a higher CTN compared with invertebrates. Surprisingly, there are still no current data sets for comparable alternative splicing indices, and controlling for transcript abundance in comparative analyses of ASP is the exception rather than the rule. The resulting lack of comparable estimates for the number of alternative splicing events per gene has hampered efforts to quantify ASP across taxa (Harrison et al. 2002), the accumulation of splicing events over time (Warnefors and Eyre-Walker 2011), and the link between alternative splicing rates and organism complexity (Nilsen and Graveley 2010; Xue et al. 2012). The only attempt to directly assess the relationship between alternative splicing variation and CTN (Schad et al. 2011) was considered inconclusive by the authors because of the lack of comparable alternative splicing measures.

Here, we assess the prevalence of alternative splicing in 47 eukaryotic genomes by calculating a comparable index of alternative splicing, which corrects for differences in transcript coverage (adapted from Kim et al. [2007]; see Materials and Methods). The species examined include metazoans, plants, fungi, and protists. We then examined how these alternative splicing indices relate to organism complexity and compared the strength of alternative splicing as a predictor of CTN to previously described correlates, including the number of protein-interacting domains encoded per gene (Xia et al. 2008), protein disorder (Romero et al. 2006; Dunker et al. 2008; Schad et al. 2011; Xue et al. 2012), the number of PPIs, gene number, and various measures of coding region length (Schad et al. 2011).

We find that alternative splicing has steadily increased over the last 1,400 My of eukaryotic evolution. We also find that alternative splicing is strongly associated with CTN and that this relationship is not a by-product of the relationship between various genomic features and complexity.

It is important to note that if increases in the proportion of alternatively spliced genes or the level of alternative splicing these genes undergo are linked with CTN, such an association would not constitute proof of causality. Under a “nonadaptive" model, the association of alternative splicing and organism complexity could be a by-product of the link between complexity and a lower effective population size (*N*_e_). The passive emergence of “genomic complexity" and even organismal complexity itself is suggested by the work of Lynch and coworkers, who argue that nonadaptive processes explain the majority of the variance in organism complexity as “more complex" organisms have a smaller *N*_e_ (Lynch and Conery 2003; Lynch 2007). As documented consequences of a comparatively small *N*_e_ include the accumulation of slightly deleterious mutations, both in coding (Nikolaev et al. 2007; Popadin et al. 2007; Gayral et al. 2013) and regulatory (Keightley et al. 2005) sequences, as well as an increase in average intron and coding region lengths (Lynch and Conery 2003), it is reasonable to expect that mutations impairing splicing regulation will accumulate more rapidly in more complex organisms resulting in higher (but not necessarily functional) transcript diversity. Consistent with this, single species studies have shown that a significant proportion of alternative splicing events are probably the result of noncoding “noise" and not biologically meaningful (Pickrell et al. 2010; Leoni et al. 2011).

Using a limited sample size, we do not find any evidence to suggest that the association of alternative splicing and CTN is explained by differences in *N*_e_. To the best of our knowledge, this is the most comprehensive assessment of alternative splicing levels (ASLs) covering all major eukaryotic taxa, and the first time in which the link between alternative splicing and CTN has been assessed using a comparative index of alternative splicing which corrects for differential transcript coverage.

## Results

### ASP Has Increased throughout Evolutionary Time

To assess whether ASLs have changed over time, over 39 million publicly available partial transcripts, representing 112 eukaryotes (20 protists, 18 plants, 23 fungi and 51 metazoans including 23 chordates), were aligned to their corresponding genomes to identify alternative splicing events (see Materials and Methods). To minimize the strong dependence of alternative splicing event detection on transcript coverage per gene (Brett et al. 2002; Kim et al. 2004; Kim et al. 2007; Takeda et al. 2008; Mollet et al. 2010; Nilsen and Graveley 2010; Schad et al. 2011), we used a transcript normalization protocol (Kim et al. 2007) where alternative splicing events are identified in randomly selected samples of 10 expressed sequence tags (ESTs) per gene. We obtained a comparable alternative splicing index per gene by averaging the number of alternative splicing events in 100 samples (Kim et al. 2007) (supplementary fig. S1, Supplementary Material online).

**Fig. 1.**
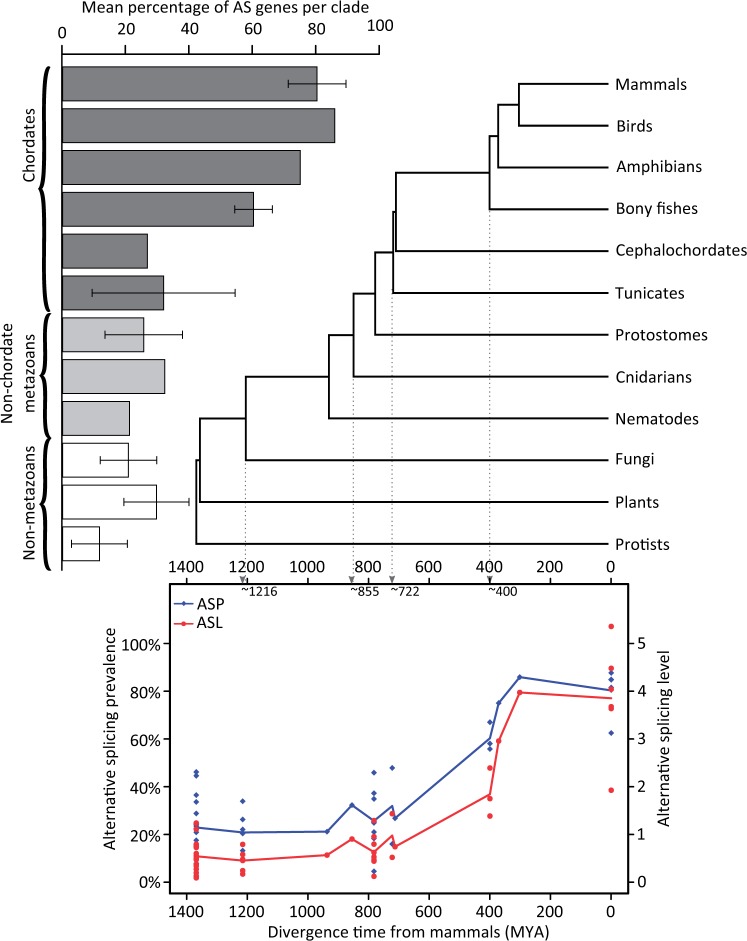
Variance in alternative splicing over evolutionary time. Bars show the average percentage of alternatively spliced genes per species grouped according to their divergence from humans, as shown in the adjacent phylogenetic tree (data from Hedges et al. 2006), and their taxonomic category (chordate, nonchordate metazoan, or nonmetazoan). The scatter plot shows changes in alternative splicing prevalance, that is, the percentage of alternatively spliced genes per genome (blue) and in alternative splicing level, that is, the average number of alternative splicing events per gene for each species (red). Trend lines represent the mean of all values at each divergence time. Although the relative positions of cephalochordates and tunicates on this tree are disputed (Delsuc et al. 2006), this does not significantly alter the trend.

Using the comparable alternative splicing index, we calculated for each species both ASP, defined as the proportion of alternatively spliced genes in the sample of genes analyzed, and ASL, defined as the average number of alternative splicing events per gene. Genomes with comparable alternative splicing estimates available for fewer than 500 genes were excluded from further analyses leaving, in total, 47 species (6 protists, 10 plants, 6 fungi, and 25 metazoans; supplementary table S1, Supplementary Material online). We found that both ASP and ASL vary among eukaryotic clades with chordates having both the highest ASP and ASL compared with nonchordate metazoans, fungi, plants, and protists (fig. 1 and supplementary table S1, Supplementary Material online). Although our ASP estimates are higher in most clades compared with a previous study based on eight species using comparable alternative splicing indices, the relative differences among clades are consistent (Kim et al. 2007).

An increase in alternative splicing through evolutionary time (fig. 1) is consistent with observations reporting links between ASP and evolutionary time restricted to metazoan species (Warnefors and Eyre-Walker 2011) and show that it is not an artifact of differential transcript coverage among species (Nilsen and Graveley 2010; Schad et al. 2011). The higher prevalence and levels of alternative splicing in plant species compared with fungi and protists suggest that AS levels have independently increased in this lineage.

Overall, by using comparable alternative splicing estimates from species covering all major eukaryotic clades and correcting for differential transcript coverage, we show that alternative splicing has increased over the last 1,400 My of eukaryotic evolution in the metazoan lineage with a more moderate and potentially independent rise in alternative splicing in plants.

### Alternative Splicing Is a Strong Predictor of Organism Complexity, Assayed as Cell Type Diversity

**Fig. 2.**
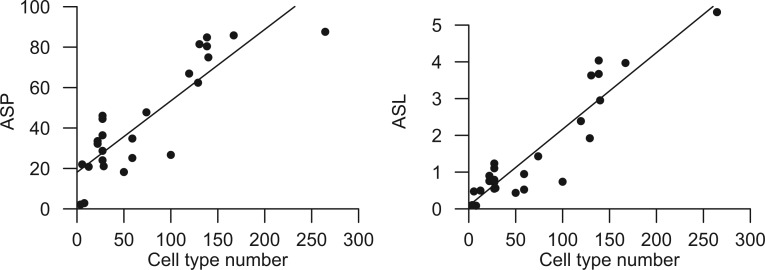
Relationship between alternative splicing and organism complexity, assayed as CTN. Graphs show the relationship between CTN and ASP (*r*^2 ^= 0.76; *P* = 9.36 × 10^−9^) and ASL (*r*^2 ^= 0.83; *P* = 1.77 × 10^−10^).

A previous attempt to assess the link between alternative splicing and organism complexity, assayed as the number of distinct cell types (Schad et al. 2011), was rendered inconclusive because of the known bias caused by differential transcript sequence coverage among genes and species (Brett et al. 2002; Kim et al. 2004; Kim et al. 2007; Takeda et al. 2008; Mollet et al. 2010; Nilsen and Graveley 2010; Schad et al. 2011). As such, we assessed the relationship of ASP and ASL with the number of distinct cell types per species (CTN) as a proxy of organism complexity using the comparable AS index (see Materials and Methods). We found that both ASL and ASP are strongly associated with CTN (ASP: *r*^2 ^= 0.76, *P* = 9.36 × 10^−^^9^; ASL: *r*^2 ^= 0.83, *P* = 1.77 × 10^−^^10^; supplementary table S2, Supplementary Material online, and fig. 2). This association remains strong when restricting the analyses to the metazoan-fungi lineage (for ASP, *r*^2 ^= 0.71, *P* = 2.45 × 10^−^^5^, and for ASL, *r*^2 ^= 0.81, *P* = 1.28 × 10^−^^6^; supplementary table S3, Supplementary Material online).

Several genomic and functional parameters have previously been associated with organism complexity (using CTN as a proxy). Xia et al. (2008) reported a strong link between CTN and PPI domain coverage. Other genomic variables found to have a more moderate association with CTN include protein disorder (Romero et al. 2006; Dunker et al. 2008; Schad et al. 2011; Xue et al. 2012) and proteome size (assayed as concatenated protein length) (Schad et al. 2011). Gene number, previously found to be unrelated to CTN, has recently been reconsidered as a significant predictor but only after plant genomes are excluded from the analyses (Schad et al. 2011).

How does alternative splicing compare to these previously reported predictors of CTN? To address this, we compared the relationship between CTN and alternative splicing with that of 12 additional genomic measures of protein interactivity as well as proteome disorder, gene length, and number, all previously linked to CTN (see Materials and Methods for descriptions and sources of each variable assessed). Of all parameters tested, ASL was found to have the strongest association with CTN (*r*^2 ^= 0.83, *P* = 1.77 × 10^−^^10^) followed by ASP and the average number of PPI domains per protein (*r*^2 ^= 0.76, *P* = 9.36 × 10^−^^9^ and *r*^2 ^= 0.64, *P* = 8.19 × 10^−^^11^ respectively; supplementary table S2, Supplementary Material online). We then re-examined the relationship between each parameter with CTN restricting the analyses to a set of 24 species for which data in all variables tested were available. The mean number of interactions per protein was not included in this or subsequent analyses due to the small number of species for which data were available (*n* = 10). ASL remained the top predictor of CTN (*r*^2 ^= 0.87, *P* = 2.80 × 10^−^^11^) with ASP showing an increased (*r*^2 ^= 0.80, *P* = 2.66 × 10^−^^9^) and the average number of PPI domains per protein a decreased association with CTN (*r*^2 ^= 0.59, *P* = 6.42 × 10^−^^6^; table 1).

**Table 1.** Association between CTN and Genomic Features Before and After Phylogenetic Signal Correction in 24 Eukaryotic Species.

Category	Variable	Linear Regression		PGLS Regression		
		*r*^2^	*P*	*r*^2^	*P*	*λ*
Alternative splicing	ASL	0.87	2.80 × 10^−11^	0.87	1.59 × 10^−13^	0
	ASP	0.80	2.66 × 10^−9^	0.77	8.38 × 10^−11^	0.05
Sizes and lengths	Number of genes	−0.01	0.40	0.26	1.23 × 10^−3^	0.76
	Average protein length	−0.05	0.97	0.12	0.03	0.79
	Proteome information content	3.25 × 10^−3^	0.31	0.09	0.05	0.65
	Proteome size	0.31	2.59 × 10^−3^	0.49	4.08 × 10^−6^	0.75
Disorder	Mean % of disordered binding sites	−0.03	0.59	0.02	0.26	0.71
	Mean number of disordered binding sites	−0.04	0.78	−0.04	0.99	0.68
	Total number of disordered binding sites	0.04	0.18	0.21	3.97 × 10^−3^	0.69
	Mean proteome disorder	−0.03	0.64	6.45 × 10^−3^	0.34	0.71
Interactivity	% PPI domain seq per protein	0.60	5.36 × 10^−6^	0.60	1.30 × 10^−7^	0
	Average number of PPI domains per protein	0.59	6.42 × 10^−6^	0.59	1.61 × 10^−7^	0
	Proportion of proteins with 1+PPI domains	0.54	2.33 × 10^−5^	0.54	7.80 × 10^−7^	0

As the relationship between genomic parameters and CTN has been shown to increase after the removal of plant genomes (Schad et al. 2011), we reassessed the predictive power of all parameters after restricting the analyses to the metazoan-fungi lineage. This resulted in a stronger association between CTN and many parameters with the two alternative splicing indices remaining the best predictors of CTN (supplementary table S3, Supplementary Material online). Consistent with previous findings (Schad et al. 2011), when plant genomes are excluded, gene number was found to be significantly associated with CTN (*r*^2 ^= 0.34, *P* = 1.74 × 10^−^^3^; supplementary table S3, Supplementary Material online).

Because of the tendency of related species to resemble one another, it is also necessary to control for this nonindependence in a comparative analysis of patterns across species. Pagel’s *λ* measures the extent to which observed correlations between traits reflect their shared evolutionary history assuming an evolutionary model under Brownian motion (Pagel 1999). For the 24 species for which data in all variables tested were available, we obtained estimates of *λ* and restricted log likelihood for the correlations between CTN and each genomic variables, recalculating each correlation to account for phylogenetic nonindependence of the variables by fitting a phylogenetic generalized least squares (PGLS) model (see Materials and Methods). ASL remained the top predictor of CTN even after taking into account the strength of the phylogenetic signal (*r*^2 ^= 0.87, *P* = 1.59 × 10^−^^13^, *λ* = 0), followed by ASP (*r*^2 ^= 0.77, *P* = 8.38 × 10^−^^11^, *λ* = 0.052) and the percentage of PPI domain sequence per protein (*r*^2 ^= 0.60, *P* = 1.3 × 10^−^^7^, *λ* = 0; table 1). This pattern holds true if we only take into account metazoan and fungal species (supplementary table S3, Supplementary Material online).

As most of the assessed parameters covary among themselves (supplementary tables S4 and S5, Supplementary Material online), the association between some variables with CTN may be secondary to their covariance with another genomic feature which is in turn linked to CTN. To identify the genomic parameters that significantly contribute to CTN, we carried out a stepwise analysis (see Materials and Methods). In the optimal stepwise regression model, the majority of the variance in CTN is explained by ASL, supported by proteome size (supplementary table S6, Supplementary Material online). Similar results are obtained when constraining the data to the metazoan-fungal lineage (supplementary table S6, Supplementary Material online). In fact, contrasting each variable directly against AS by including ASL/ASP in multiple regression models with each additional variable revealed that in all cases, only the AS parameter remained significantly associated with CTN (supplementary table S2, Supplementary Material online). The only exception was proteome size that remained significantly associated with CTN after correcting for either ASP or ASL, but only when fungi and metazoans were included in the analysis (supplementary table S3, Supplementary Material online).

**Fig. 3.**
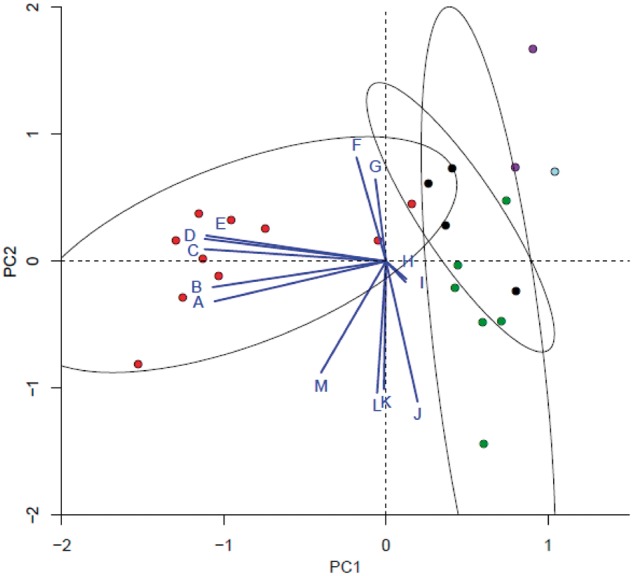
Biplot of the first two principal components built from 13 functional genomic variables available for 24 species (see supplementary table S1, Supplementary Material online). Graph shows the distribution of species along PC1, which explains 35.2% of the variance in this data set, and PC2, which accounts for 31.4%. Points represent each of 24 species for which data were available for all variables and are colored by taxonomic category: chordates (red), nonchordate metazoans (black), plants (green), fungi (blue), and protists (purple). Ellipses show the clustering of species according to their dispersion along PC1 and PC2 (with confidence limit 0.95). Blue lines radiating from (0,0) represent each variable included in the analysis. The direction of each line represents the highest correlation coefficient between the PC scores and the variable, with the length of each line proportional to the strength of this correlation. Letter codes for each variable: ASL (A), ASP (B), % PPI domain sequence per protein (C), proportion of proteins with at least one PPI domain (D), average number of PPI domains per protein (E), average protein length (F), mean number of disordered binding sites per protein (G), mean proteome disorder (H), mean % of disordered binding sites per protein (I), number of genes (J), total number of disordered binding sites per proteome (K), proteome information content (L), and proteome size (M).

To best capture the predictive value of sets of covarying variables, we used a principal component analysis to reduce the dimensionality among the 13 predictors of complexity. This analysis was performed on a subset of species where data were available for all predictors (*n* = 24). Interestingly, PC1 and PC2 (which explain 35.2% and 31.4% of the variance in the matrix, respectively) allow chordates to be differentiated from all other species (fig. 3). Of all resulting principal components, we found that PC1 is the only significant predictor of CTN (*r*^2 ^= 0.66, *P* = 8.58 × 10^−^^7^). The two alternative splicing variables (ASP and ASL) and the three protein interactivity variables (average number of PPI domains per protein, PPI domain coverage, and the proportion of proteins with at least one PPI domain) were found to be the main contributors to PC1. Similar results were obtained when restricting the analyses to the metazoan-fungi lineage (data not shown). It is worth noting, however, that the value of *r*^2^ when regressing PC1 against CTN, when including either all species or only metazoans and fungi, is lower than that of ASL (*r*^2 ^= 0.83, *P* = 1.77 × 10^−^^10^), suggesting that collapsing the dimensionality of the variables does not improve the prediction of CTN beyond the variance explained by ASL alone.

The above results show that AS is significantly associated with CTN and that this association is not explained as a by-product of the relationship between AS and other genomic features also related to CTN. However, it is possible that some of these associations might be explained by ascertainment bias resulting from the fact that humans and other closely related species have been disproportionately studied. With the exceptions of *Caenorhabditis elegans* and *Drosophila melanogaster*, larger amounts of data exist for vertebrates than other species. It is possible that the higher estimates of AS and other genomic features, and even higher CTN among vertebrates, might partly result from the greater availability of data for these species. To address this possibility, we used the total number of ESTs per species as a proxy for interest in a species as higher transcript availability has a direct impact on the quality of genome annotation. Compared with other proxies of “research interest" such as “number of publications per species," the number of ESTs approximates how much data have accumulated rather than how many interpretations of it there have been.

We established that the number of ESTs per species is significantly associated with various genomic characteristics (supplementary table S7, Supplementary Material online). Notably, ASL and ASP, as well as CTN, were found to be significantly related with transcript number per species (ASL: *r*^2 ^= 0.45, *P* = 7.29 × 10^−^^7^; ASP: *r*^2 ^= 0.39, *P* = 8.01 × 10^−^^6^; complexity *r*^2 ^= 0.41, *P* = 5.01 × 10^−^^5^). Thus, we re-examined the relationship of CTN with AS and other gene features using the residuals of a quadratic polynomial regression with EST number. This correction resulted in a marked reduction in the variance in CTN explained by ASL and ASP (*r*^2 ^= 0.47, *P* = 9.84 × 10^−^^5^ and *r*^2 ^= 0.57, *P* = 8.82 × 10^−^^6^, respectively; supplementary table S8, Supplementary Material online). Correcting all variables by transcript coverage also reduced the predictive value of other gene features for CTN (supplementary table S8, Supplementary Material online). However, the relative order of gene feature parameters as predictors of CTN remained unaltered with splicing and, to a lesser extent, the degree of protein–protein interactivity the most strongly associated with CTN (supplementary table S8, Supplementary Material online). Furthermore, if considering all 13 variables, the optimal stepwise regression model (see Materials and Methods) explained 90% of the variance in CTN (*P* = 1.81 × 10^−^^5^), with the strongest of five predictors being ASP (supplementary table S9, Supplementary Material online). When restricting the analyses to the fungi-metazoan lineage, we found that the optimal regression model contained only two regressors, ASP and the mean percentage of disordered binding sites per protein (see Materials and Methods for a description of this variable) (supplementary table S9, Supplementary Material online). In fact, only three parameters (average protein length, the number of genes, and the total number of disordered binding sites per protein) remained significantly associated with CTN in a regression model directly comparing each variable with either ASP or ASL (supplementary table S8, Supplementary Material online). An alternative transformation of the data, taking the natural log of EST number, resulted in lower correlation coefficients, but the relative strength of each variable in a regression against complexity remained unchanged (supplementary table S10, Supplementary Material online).

Our data span a diverse range of species with associated variations in the number of available ESTs per species (supplementary table S1, Supplementary Material online). For genomes with lower EST numbers (often those that also have a lower CTN), highly expressed genes will make a disproportionate contribution to each species’ comparative alternative splicing index as the number of genes with the minimum required number of ESTs will be smaller. As such, we expect lowly expressed genes to primarily contribute data for genomes with a higher number of available ESTs.

Under the nonadaptive model, a reduced *N*_e_ among more complex organisms (assayed as those with higher CTN) would result in an accumulation of mutations detrimental to splicing regulation, potentially resulting in the proliferation of “noisy" alternative splicing events. Such neutral increases in alternative splicing should be particularly pronounced among lowly expressed genes, which, on average, are under lower selective pressures compared with highly expressed genes. Importantly for this study, if lowly expressed genes are more highly spliced, then our data would overestimate ASL for species with high EST numbers, artificially inflating the correlation strength with CTN.

Using microarray data for four model species (human, mouse, *Caenorhabditis elegans,* and *Arabidopsis thaliana*; see Materials and Methods), we find that, as expected, there is a strong correlation between the number of ESTs per gene and gene expression level. However, contrary to the prediction of the nonadaptive model, we found that the more highly expressed genes are also more highly spliced (supplementary figs. S2–S5, Supplementary Material online). Therefore, our data might be underestimating ASP and ASL in genomes with a higher number of available ESTs, as more lowly expressed genes—with lower ASLs—disproportionately contribute to the species’ alternative splicing indices. By extension, the relationship of AS with CTN might also be underestimated.

## Discussion

Here, we have assessed ASLs in 47 eukaryotic species and showed that alternative splicing has increased over the last 1,400 My of evolution. Our data range from *Plasmodium falciparum*, in which 3% of genes are spliced with an average of 0.09 splice events per gene, to humans, where 88% of genes are spliced with an average of 5.35 splice events per gene. Consistent with the findings of Kim et al. (2007), we find that chordates have higher levels of alternative splicing than any other taxonomic group with mammals and birds having both proportionately more genes that are alternatively spliced (ASP) and a higher number of alternative splicing events per gene (ASL). We observed significant increases over time in ASP and ASL for the opisthokonts and show that past claims for an increased level of alternative splicing along the evolution of metazoans are not explained by differential transcript coverage (Warnefors and Eyre-Walker 2011). Our data do not support a previous claim for lower ASLs among birds compared with mammalian species (Chacko and Ranganathan 2009), and in fact, ASLs in the chicken genome were found to be among the highest of all species tested.

Plant genomes were found to have higher levels of alternative splicing than both protist and fungal species, comparable to those found among invertebrate species. This is consistent with relatively low levels of alternative splicing in the eukaryotic ancestor with independent rises in the plant and metazoan lineages. None of the plant genomes we examined, however, match the levels of alternative splicing observed in the vertebrate lineage.

Our results demonstrate a strong association between alternative splicing and organism complexity providing, to the best of our knowledge, the first systematic evidence for a link between these two variables. In this study, we have used the number of cell types as a proxy for organism complexity. CTN has been proposed as an indicator of an organism complexity as the higher number of components or cell types in more complex organisms should reflect, to some degree, their higher number of functions (McShea 2000). We acknowledge, however, that complexity is difficult to define and even more difficult to measure and that all operational definitions for “complexity" are, to various degrees, contentious (Adami 2002). Several proxies of organismal complexity have been proposed; however, these measures are either relevant to some taxonomic groups, such as encephalization coefficient, or no measurements are available for a large number of species, such as phenotypic complexity (Tenaillon et al. 2007). Although accepting that “organism complexity" is likely to be a multidimensional variable encompassing many other features, we chose this measure as, compared with other proxies, cell types are more easily quantifiable for organisms from distant taxonomic groups. It is important to note that, as CTN data are drawn from a diverse range of studies (see Materials and Methods), more detailed characterizations of CTN can appear anomalous. For example, we expect chimpanzees to have a similar CTN to humans, but currently, humans are the better characterized species and as such the human CTN appears higher (supplementary table S1, Supplementary Material online). To address whether this type of outlier confounds our results, we repeat our analyses using the average CTN for the order each species belongs to. This makes the assumption that any variation in CTN between species of a given order reflects measurement noise, rather than relevant biological information. Our results do not significantly differ when using these alternate values of CTN (supplementary tables S11 and S12, Supplementary Material online).

Importantly, as most past studies analyzing the relationship between various genomic features and organism complexity have adopted CTN as a proxy (Xia et al. 2008; Chen et al. 2011; Schad et al. 2011; Xue et al. 2012), its use allowed us to contrast our results with those of others. Such comparisons showed that the relationship of alternative splicing and CTN is not secondary to other genomic features previously associated with CTN, including proteome size (measured as total protein coding sequence length [Schad et al. 2011]), protein disorder (Schad et al. 2011; Xue et al. 2012), and protein interactivity.

Before the full sequencing of nuclear eukaryotic genomes became widespread, gene number was expected to have a direct relationship with organism complexity as more genes would encode a higher number of proteins boosting the number of potential molecular interactions (Romero et al. 2006; Dunker et al. 2008). The sequencing of the human genome, however, found no evidence for such an association (Fields et al. 1994). The discrepancy between organism complexity and gene content became known as the G-paradox (Claverie 2001; Betran and Long 2002; Hahn and Wray 2002; Taft and Mattick 2003). However, a recent study concluded that gene number and organism complexity are related after all, albeit only when plant species are removed from the analyses (Schad et al. 2011).

Our findings also support a significant association between gene number and CTN in the absence of plant genomes (*r*^2 ^= 0.34, *P* = 1.74 × 10^−^^3^; supplementary table S3, Supplementary Material online). However, ASL has a stronger association with CTN (*r*^2 ^= 0.77, *P* = 1.09 × 10^−^^8^) and is sufficient to explain the relationship between CTN and gene number.

Unlike alternative splicing and gene number, which directly impact on the number of interacting proteins, additional gene features linked to CTN can boost the interactivity potential of individual proteins without expanding their number. One of the simplest measures of the functional potential of the proteome, total coding region length, has been found to be significantly associated with CTN (Schad et al. 2011). Although we observed a similar association between proteome size and CTN, this relationship is entirely explained as a by-product of both variables’ covariance with alternative splicing. Proteome size remains a marginal, albeit significant, predictor of CTN in a stepwise regression model restricted to the metazoan and fungi lineage where ASL was the strongest variable (table 1). Moreover, proteome size was not a significant contributor to the only principal component found to be significantly associated with CTN.

Protein disorder—the lack of equilibrium in a protein’s 3D structure under physiological conditions (Romero et al. 2006)—has been proposed as a candidate predictor of organism complexity as higher intrinsic disorder allows individual proteins to adopt a greater variety of conformations, increasing the average number of interacting partners per protein and potentially boosting functional diversification of the proteome (Romero et al. 2006; Dunker et al. 2008). Nevertheless, subsequent findings show the association between disorder and CTN only explains any substantial amount of variance when bacterial species are included (Schad et al. 2011; Xue et al. 2012). Our analyses of protein disorder using both stepwise regressions and principal component analysis do not provide any evidence of hidden covariance between protein disorder and CTN. Moreover, despite the fact that past studies have found a higher than expected number of disordered motifs in alternatively spliced areas at the gene level (Romero et al. 2006; Buljan et al. 2012), we do not find a significant association between protein disorder and alternative splicing per species (supplementary tables S4 and S5, Supplementary Material online).

Finally, a third measure of potential molecular interactions per protein, the presence of PPI domains, has been shown to be strongly associated with CTN (Xia et al. 2008). We found three protein interactivity parameters—PPI domain coverage, the average number of PPI domains per protein, and the proportion of proteins with at least one PPI domain—to be significantly associated with CTN regardless of the set of species examined (supplementary tables S2 and S3, Supplementary Material online). A head-to-head comparison between predictors of CTN showed that protein interactivity measures are better predictors of CTN than any other variable with the exception of alternative splicing. After controlling for alternative splicing, however, no protein interactivity parameter was found to be significantly associated with CTN (supplementary tables S2 and S3, Supplementary Material online). An additional measure of protein interactivity previously associated with CTN, the mean number of PPIs (Schad et al. 2011), was not included in most of our analyses as data were limited to only 10 species in our set. These comparisons show that although protein interactivity is significantly associated with CTN, there is a great overlap between the variance in CTN explained by protein interactivity and that explained by alternative splicing.

Several studies have proposed an association between alternative splicing and protein domain content, suggesting that alternative splicing could act as a buffer against reduced functionality because of “domain overload"—too many protein domains or domains in the wrong combination (Kriventseva et al. 2003; Resch et al. 2004; Floris et al. 2008). A large-scale analysis has shown that protein domains are nonrandomly combined in functional proteins with fewer protein domain co-occurrences observed than expected, suggesting that certain protein domains “avoid" each other (Parikesit et al. 2011), whereas other domains—including PPI domains—are “promiscuous" and tend to coexist within individual transcripts (Basu et al. 2008). Our analyses of covariance among functional gene variables showed that alternative splicing and PPI measures are positively correlated—genomes with higher levels of alternative splicing also have a higher PPI domain presence. We further examined the association between ASL and PPI domain coverage within species but found only a marginal association between the two variables constrained to a few species (supplementary table S13, Supplementary Material online). This finding suggests that although genomes with a high level of alternative splicing also tend to have a higher PPI domain coverage, there is no support for a role for alternative splicing acting as a buffer of PPI domain overload.

Overall, our results are consistent with a direct association between alternative splicing and CTN, one which is not explained by other genomic features previously associated with organism complexity. This finding is, in principle, consistent with previous suggestions that alternative splicing may underlie the rise in complexity during eukaryotic evolution thanks to its potential to expand transcript diversity and thereby increase the number of potential molecular interactions and functions (reviewed in Xing and Lee 2007; Nilsen and Graveley 2010; Chen et al. 2012).

Nevertheless, it is important to note that the rise in CTN has been accompanied by a reduction in effective population size (Lynch and Conery 2003). Classical nearly neutral theory proposes that as effective population sizes diminish so too does the efficiency of purifying selection, resulting in the accumulation of slightly deleterious mutations, both in coding (Nikolaev et al. 2007; Popadin et al. 2007; Gayral et al. 2013) and regulatory (Keightley et al. 2005) sequences. The increased role of drift relative to selection has also been invoked to explain the proliferation of a number of genomic features among increasingly complex species (Lynch and Conery 2003; Lynch 2007). Although more recent studies have disputed this conclusion (Kuo et al. 2009; Whitney and Garland 2010; Whitney et al. 2011), a significant proportion of alternative splicing events have nevertheless been suggested to result from noisy alternative splicing (Sorek et al. 2004; Pickrell et al. 2010; Leoni et al. 2011). Thus, it is possible that the observed increase in alternative splicing among more complex species might be the result of increased genetic drift as a result of reductions in effective population size, rather than being directly associated with organism complexity. Using estimates of effective population size for the 12 species represented in this study (Lynch and Conery 2003), we found that a genome’s capacity for alternative splicing remains strongly correlated with CTN even after controlling for effective population size (partial Spearman’s correlation coefficients: ASL = 0.71, *P* = 2.37 × 10^−^^3^; ASP = 0.70, *P* = 3.35 × 10^−^^3^). Although based on a small sample of species, this finding suggests that the association between CTN and alternative splicing is not a by-product of reduced effective population sizes among more complex species. Future studies should be able to assess the functional contribution of increases in alternative splicing in the eukaryotic lineages we report here.

In addition, it is worth noting that a significant correlation of any genomic feature with CTN does not necessarily demonstrate a causal role on the evolution of organism complexity, that is, a higher CTN. It is beyond the scope of this study to address this directly. Nevertheless, network theory provides some clues, which allows us to speculate as to the likelihood that increases in transcript diversification, facilitated by alternative splicing, have affected the evolution of organism complexity. Boolean networks have been proposed as models for genetic networks as the attractors, representing different stable patterns of gene expression, correspond to different cell types (Kauffman 1969; Serra et al. 2010). In Boolean networks, increases in the number of nodes leads to a higher number of attractors within the network at a rate equal to or exceeding the square root of the number of nodes in the network (Samuelsson and Troein 2003). If we imagine each distinct transcript as a node in the genetic network, we can speculate that alternative splicing, by increasing the number of nodes (transcripts), would lead to an increased number of attractors (cell types). Indeed, a previous study that generated relational networks for seven species associated the number of functions in a proteome with the number of polyform transcriptional units in the genome, those that produce protein isoforms with different functional assignments (which are strongly associated with the levels of splicing). Various properties of these networks (such as the number of nodes) were found to be strongly associated with organism complexity, suggesting a link between splicing and both multifunctionality and multicellularity (Kanapin et al. 2010).

We conclude that alternative splicing increases over the last 1,400 My of eukaryotic evolution are strongly associated with CTN. Furthermore, this association is stronger and more robust than other parameters previously associated with CTN, although we cannot rule out the contributions of other genomic features as many covary. Our findings are consistent with an adaptive scenario whereby a genome’s capacity for alternative splicing—with its resulting expansion of the transcript pool—could constitute a critical component of the underlying mechanisms explaining the diversification of cell types and the rise in organism complexity over time. Nevertheless, the data here presented do not allow us to reach a conclusion on the functional relevance of increases in alternative splicing or to establish causality regarding the association of alternative splicing and organism complexity; thus, it is possible that a “nonadaptive model" may account for it.

To the best of our knowledge, our results represent the first systematic assessment of the relationship between alternative splicing, evolutionary time, and CTN and provide evidence for a strong association of alternative splicing and CTN. Our results further constitute the most comprehensive head-to-head comparison, to date, of variables associated with CTN.

## Materials and Methods

### Organism Complexity

The number of unique cell types was used as a proxy of organism complexity. Estimates of CTN per species were compiled from previous studies (Valentine et al. 1994; Bell and Mooers 1997; Hedges et al. 2004; Haygood and Investigators 2006; Lang et al. 2010; Schad et al. 2011); data in graph form from Valentine et al. (1994) as interpreted by both Erwin (2009) and Vogel and Chothia (2006) were also included. Following the methodology of Vogel and Chothia (2006), where more than one estimate of CTN was available for a species, the average of the minimum and maximum number was used. In addition, we included a revised CTN estimate for humans (Vickaryous and Hall 2006). Supplementary table S1, Supplementary Material online, provides averaged complexity estimates for both pro- and eukaryotic species, whereas supplementary table S14, Supplementary Material online, shows the sources.

### Identification of Alternative Splicing Events

Comparable alternative splicing events were obtained using the following approach*.* Over 39 million EST sequences, accounting for 112 species, were downloaded from dbEST (Boguski et al. 1993) and matched to their corresponding genome using GMAP (Wu and Watanabe 2005) (these species are identified in supplementary table S1, Supplementary Material online, by a positive value in the column titled “total number of ESTs”). Genome sequences and annotations were obtained from sources contained in supplementary table S1, Supplementary Material online. Cancer-derived EST libraries from human and mouse were removed from all analyses presented. To ensure high-quality alignments, we only retained those ESTs with 95% identity. ESTs were assigned to genes using gene annotation coordinates. EST alignments were then used to create an exon template. These templates were generally in agreement with existing exon annotations and also identify a small number of nonannotated exons and discard orphan exons likely to be nested genes. Alternative splicing events per gene were identified by comparing alignment coordinates for each individual EST to exon annotations. A comparable alternative splicing index that avoids transcript coverage biases was obtained using the transcript normalization method described by Kim et al. (2007). Briefly, for each gene with greater than 10 ESTs, 100 random samples of 10 ESTs were selected. The number of alternative splicing events were calculated for each random sample (as detailed earlier), with an overall average calculated per gene. The ability of this method to correct for transcript coverage bias and calculate an accurate number of alternative splicing events is shown in supplementary figure S1, Supplementary Material online. To estimate ASP, a gene was considered to be alternatively spliced if it had at least an average of one alternative splicing event identified in each of the 100 random samples.

### Additional Functional Genomic Parameters

Gene number per species was obtained from Ensembl BioMart version 0.8 (March 2013) (Kinsella et al. 2011). Proteome size (total amino acids encoded by all peptides), proteome information content (total amino acids encoded by primary transcripts only), and average protein length were calculated from mRNA transcripts obtained from Ensembl BioMart version 0.8 (March 2013) (Kinsella et al. 2011). The exception is the lancelet, *Branchiostoma floridae*, where transcripts were obtained from Putnam et al. (2008). PPI domains per protein were identified using HMMER3 with default parameters (Finn et al. 2011) and the Pfam-A database (Finn et al. 2008), with results parsed to consider matches to the 642 confirmed PPI domains as described by Xia et al. (2008). Protein disorder data were obtained from Schad et al. (2011). “Disordered sites" are those which are not at equilibrium in the protein’s 3D structure under physiological conditions and can thus adopt a greater variety of conformations. We obtained the mean number of disordered binding sites per protein, the total number of disordered binding sites across all annotated proteins per species, and the mean percentage of disordered binding sites per protein (i.e., the mean number of disordered sites per protein as a percentage of the protein’s length). The latter term is considered the disorder of the protein. Mean proteome disorder is taken as the mean disorder per protein. The average number of PPIs per protein for each species was also obtained from Schad et al. (2011). Data on effective population size were obtained from Lynch and Conery (2003).

### Statistical Analysis

All statistical tests were performed in R, version 2.15.2 (Team 2012). For stepwise regression analysis, new regressors are included at each step according to the Akaike Information Criterion (Akaike 1974), estimated using the package “MASS" (Venables and Ripley 2002). Principal component analysis was performed using the R packages “FactoMineR" (Lê et al. 2008) and “Vegan."

### Correction for Phylogenetic Autocorrelation

To assess and control for the strength of the phylogenetic signal on the correlation between CTN and the different genomic variables in this study, we computed Pagel’s *λ* (Pagel 1999) based on maximization of the restricted log-likelihood using the gls subroutine from the R-package nlme (Pinheiro et al. 2013). Optimum negative values of Pagel’s *λ* are reported as *λ* = 0. We used the subroutine PGLS in the R-package Caper (Orme et al. 2012) to examine the “true" associations between the different genomic variables and CTN after using the optimal *λ* values to control for the strength of the phylogenetic signal. This method implements generalized least squares models, which account for phylogenetic nonindependence by incorporating the covariance between taxa into comparisons that determine the correlation between dependent and independent variables. PGLS is an extension of the independent contrasts methods proposed by Felsenstein (1985) that provides a more general and flexible approach for assessing correlations between traits while accounting for phylogenetic divergence. An ultrametric phylogenetic tree for the analyzed species was created by obtaining the divergence time between each pair of species from Hedges et al. (2006).

### Expression Level

Microarray data for four species (*Homo sapiens*, *Mus musculus*, *A. thaliana,* and *C. elegans*) were obtained from the following sources. For *H. sapiens* and *M. musculus*, Affymetrix array data analyzed by Su et al. (2004) was obtained from BioGPS (http://biogps.org, last accessed November 21, 2013). For *H. sapiens*, we obtained the expression of 11,449 genes across 28 tissues. We summarized gcRMA (GC robust multiarray average) normalized probe intensity levels to Ensembl IDs corresponding to protein coding genes. All probes matching to more than one Ensembl gene ID were removed. Expression values were then normalized against the total signal level in each tissue. For *M. musculus*, we obtained 9,825 genes with one-to-one orthologs in the human across 79 different tissues and cell types. Where more than one array exists for a given tissue, data were averaged. The per probe expression signal was summarized to Ensembl gene IDs using the average expression of all the probe sets matching a single Ensembl ID. All probes matching to more than one Ensembl gene ID were removed. Expression values were then normalized against the total signal level in each tissue. For *A. thaliana*, data were obtained from the Arabidopsis Development Atlas, as generated by the AtGenExpress Consortium (Schmid et al. 2005) (NASCARRAYS reference numbers 149–154, together representing 79 tissues, were downloaded from NASC AffyWatch [http://affymetrix.arabidopsis.info/, last accessed November 7, 2011]). Expression level was then quantified as the average gcRMA across all 79 tissues (with each value itself the mean of triplets) (Yang and Gaut 2011). For *C. elegans*, tissue-specific expression for 13 tissues (germline, hypodermis, intestine, muscle, neurons, pharynx, coelomocytes, distal tip, excretory cells, spermatheca, spermatheca uterine valve, uterus, and vulva) was obtained from Chikina et al. (2009) (http://worm-tissue.princeton.edu, last accessed November 28, 2013), who analyzed a compendium of 916 microarray experiments from 53 data sets. Expression values in this data set are already normalized to have mean 0 and variance 1. Expression level is taken as the mean across all tissues.

## Supplementary Material

Supplementary tables S1–S14 and figures S1–S5 are available at *Molecular Biology and Evolution* online (http://www.mbe.oxfordjournals.org/).

Supplementary Data
