# Supplementary material for: Correcting for Differential Transcript Coverage Reveals a Strong Relationship between Alternative Splicing and Organism Complexity
Source: Mol Biol Evol. 2014 Mar 27;31(6):1402–13. doi: 10.1093/molbev/msu083 (PMC4032128; doi:10.1093/molbev/msu083)
Supplement: Supplementary Data [file supp_31_6_1402__index.html]

Correcting for Differential Transcript Coverage Reveals a Strong Relationship between Alternative Splicing and Organism Complexity — Correcting for Differential Transcript Coverage Reveals a Strong Relationship between Alternative Splicing and Organism Complexity — Supplementary Data 

# Correcting for Differential Transcript Coverage Reveals a Strong Relationship between Alternative Splicing and Organism Complexity

## Supplementary Data

files

**Files in this Data Supplement:**

- Supplementary Data - pdf file
- Supplementary Data - pdf file
- Supplementary Data - pdf file
- Supplementary Data - pdf file
- Supplementary Data - pdf file
- Supplementary Data - xlsx file
