# Supplementary material for: Correcting for Differential Transcript Coverage Reveals a Strong Relationship between Alternative Splicing and Organism Complexity
Source: Mol Biol Evol. 2014 Mar 27;31(6):1402–13. doi: 10.1093/molbev/msu083 (PMC4032128; doi:10.1093/molbev/msu083)
Supplement: Supplementary Data [file supp_msu083_Supplementary_Figure_1.pdf]

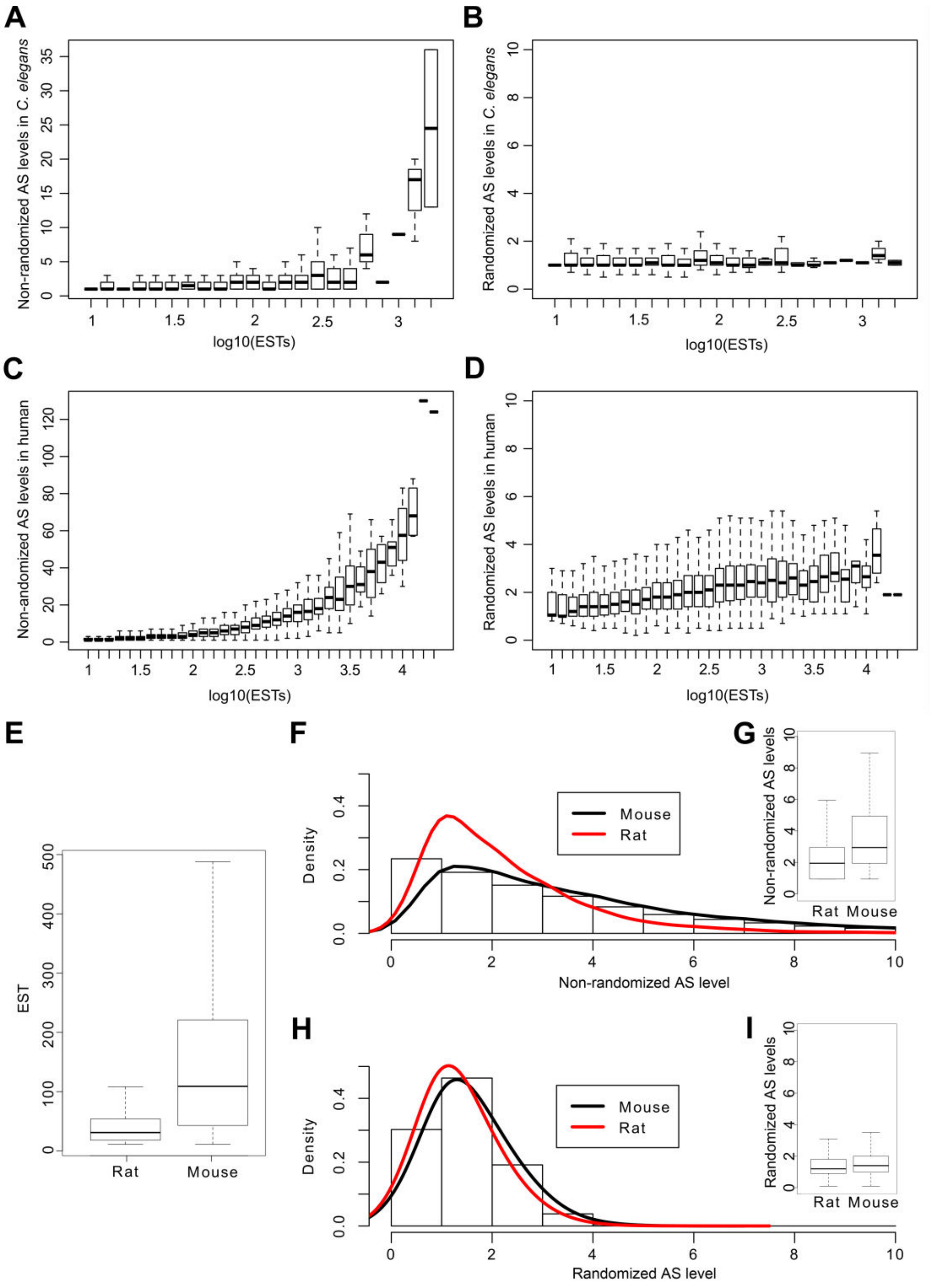

Supplementary Figure 1. Total transcript number influences alternative splicing level (ASL) detection but this bias can be corrected using a sampling method. ASL detection in genes divided by transcript coverage is shown for the nematode (A and B) and human (C and D) using both the full transcript dataset (A and C) and the

random sampling method (B and D). Large differences in the average EST coverage for both rat and mouse (E) lead to correspondingly large differences in ASE detection for the two species (F). These are greatly reduced by the use of a sampling method (H). Inset panels G and I show the average ASL in both species using both the full transcript dataset and the random sampling method, respectively.
