# Supplementary material for: Correcting for Differential Transcript Coverage Reveals a Strong Relationship between Alternative Splicing and Organism Complexity
Source: Mol Biol Evol. 2014 Mar 27;31(6):1402–13. doi: 10.1093/molbev/msu083 (PMC4032128; doi:10.1093/molbev/msu083)
Supplement: Supplementary Data [file supp_msu083_Supplementary_Figure_3.pdf]

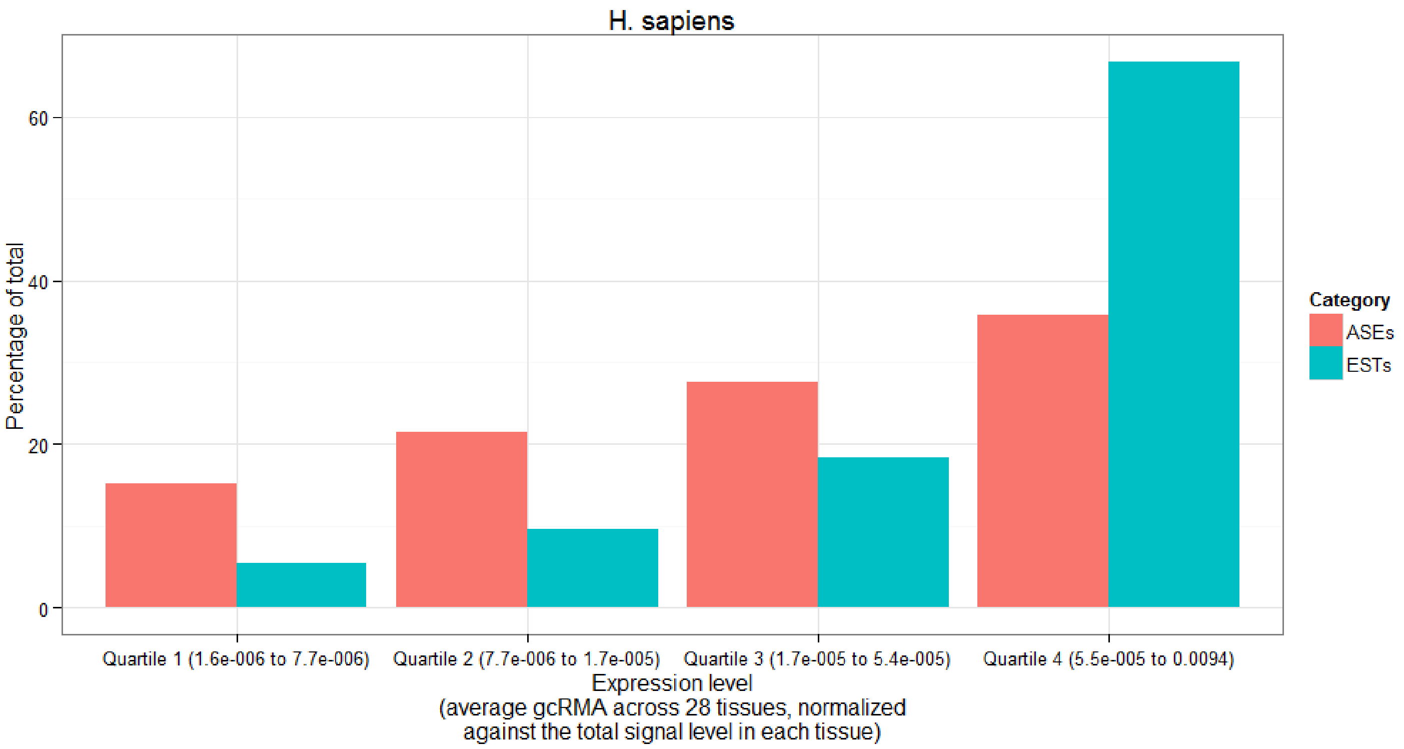

Supplementary Figure 3. Distribution of both alternative splicing events (n = 76,699) and ESTs (n = 4,510,520) for the genome of *H. sapiens*, according to the expression level of the associated gene. Expression data is partitioned into quartiles each of size n = 1618. Correlations of the number of alternative splicing events and the number of ESTs against expression level are significant in both cases (Spearman's *rho* = 0.35, $p < 2.2e^{-16}$ and *rho* = 0.53, $p < 2.2e^{-16}$).
